# Circulating Apolipoprotein B-48 as a Biomarker of Parenteral Nutrition Dependence in Adult Patients with Short Bowel Syndrome

**DOI:** 10.3390/nu15183982

**Published:** 2023-09-14

**Authors:** Salma Fourati, Annick Hamon, Rita Daclat, Joe-Elie Salem, Katell Peoc’h, Johanne Le Beyec, Francisca Joly, Jean-Marc Lacorte

**Affiliations:** 1 Service de Biochimie Endocrinienne et Oncologique, Pitié-Salpêtrière Hospital, Assistance Publique-Hôpitaux de Paris, UMR-S 1149 Centre de Recherche sur l’Inflammation Inserm, Paris Cité University, 75013 Paris, France; 2 Department of Gastroenterology, IBD and Nutrition Support, CRMR MarDi, Beaujon Hospital, Assistance Publique-Hôpitaux de Paris, 92110 Clichy, France; 3 UMR_S1166, Research Institute of Cardiovascular Disease, Metabolism and Nutrition Inserm, Pitié-Salpêtrière Hospital, Sorbonne University, 75013 Paris, France; 4 Department of Pharmacology and Clinical Investigation Centre (CIC-1901), Pitié-Salpêtrière Hospital, Assistance Publique-Hôpitaux de Paris, Sorbonne University, INSERM, 75013 Paris, France; 5 Department of Biochemistry, CRI INSERM UMR1149, HUPNVS, Assistance Publique-Hôpitaux de Paris, Paris Cité University, 75018 Paris, France; 6 Service de Biochimie Endocrinienne et Oncologique, Pitié-Salpêtrière Hospital, Assistance Publique-Hôpitaux de Paris, UMR-S 1149 Centre de Recherche sur l’Inflammation Inserm, Sorbonne University, 75013 Paris, France; johanne.le-beyec@inserm.fr; 7 Department of Gastroenterology, IBD and Nutrition Support, CRMR MarDi, Beaujon Hospital, Assistance Publique-Hôpitaux de Paris, 92110 Clichy, UMR-S 1149 Centre de Recherche sur l’Inflammation Inserm, Université Paris Cité, 75018 Paris, France; francisca.joly@aphp.fr; 8 Service de Biochimie Endocrinienne et Oncologique, Pitié-Salpêtrière Hospital, Assistance Publique-Hôpitaux de Paris, Research Unit on Cardiovascular and Metabolic Disease, UMR ICAN, Sorbonne University, Inserm, 75013 Paris, France; jean-marc.lacorte@aphp.fr

**Keywords:** parenteral nutrition, short bowel syndrome, serum apolipoprotein B-48

## Abstract

Short bowel syndrome (SBS) is a rare but serious condition that may lead to chronic intestinal failure. Citrulline concentrations are currently used to reflect the residual intestinal mass in patients with SBS, although this method has several limitations. In a cohort of patients with SBS, we quantified apolipoprotein B-48 (ApoB-48), which is exclusively synthesized by enterocytes and secreted associated with dietary lipids and investigated the relationship between ApoB-48 and clinical and biological data as well as PN dependence. A total of 51 adult patients were included, 36 of whom were PN-dependent. We found a robust positive correlation between circulating ApoB-48 and residual small bowel length, which was also found in the subgroup of patients with jejunocolic anastomosis. Fasting ApoB-48 levels were significantly lower in PN-dependent patients than in PN-weaned patients and negatively correlated with parenteral nutrition dependence. Our results suggest that ApoB-48 could be proposed as a marker of intestinal absorptive function and could be an interesting follow-up marker in patients with SBS.

## 1. Introduction

Short bowel syndrome (SBS) is a rare condition that results from an extensive surgical resection of the small intestine with partial or total colectomy. The most common pathologies leading to SBS in adults are Crohn’s disease, mesenteric ischemia, radiation enteritis, and postoperative adhesions and complications [1]. After massive resection, the remaining intestinal surface cannot absorb enough macronutrients, micronutrients, and water, leading to intestinal failure (IF). Parenteral nutrition (PN) is then the reference treatment to compensate for the resulting underenteral nutrition. A functional classification of IF has been described by ESPEN with three types based on onset, metabolism, and outcome [1,2]. The acute phase begins immediately after the surgery and lasts for days to a few weeks. Metabolically unstable patients who require complex multidisciplinary care and intravenous supplementation for prolonged periods, up to a few months, are identified as having a prolonged acute condition. The chronic phase refers to metabolically stable patients requiring long-term intravenous supplementation [3,4]. PN dependence in SBS patients is related to the remaining bowel anatomy and its residual functional capacity [5,6]. In fact, three anatomical subtypes of SBS can be identified: end-jejunostomy (type I), jejunocolic anastomosis (type II), and jejunoileal anastomosis (type III) with both the ileocecal valve and the entire colon in continuity [2].

Successful rehabilitation of patients is based on a combination of both oral and PN, as well as pharmacological treatments [7,8] tending to increase the absorptive capacity of the remaining intestine in order to reduce or wean patients from PN, which will improve their quality of life [9]. Therefore, monitoring the functional absorptive capacity of the remaining small intestine is critical for adjusting treatment and PN support.

To date, the reference method for assessing intestinal absorptive capacity is based on calculating the difference between caloric intake and fecal energy losses. These are measured by bomb calorimetry using duplicate meals and by collecting leftovers of served meals and stool output over 3 to 4 days. However, this method is not routinely used because it is too complex to implement [10,11]. Plasma citrulline, a nonessential amino acid produced by enterocytes from different precursors [10,11,12] and converted to arginine by the kidney [10], is commonly used as a biomarker of enterocyte mass and intestinal absorption in patients with SBS [13]. However, citrulline levels increase in renal failure [13], making its values uninterpretable in this context. In addition, citrulline should be used with caution in case of inflammation or severe illness [14]. Recent data suggest that disease characteristics, which contribute to the heterogeneity in patients with SBS, may also influence the efficacy of citrulline as a generalizable biomarker in IF-associated SBS [14].

Interestingly, apolipoprotein B-48 (ApoB-48), a chylomicron structural protein, is exclusively synthesized and secreted by the enterocytes. During meal absorption, lipids are transferred to the endoplasmic reticulum, where triglycerides, cholesterol, and phospholipids are associated with ApoB-48 to form a prechylomicron [15]. The maturation of the prechylomicron in the cis-golgi is associated with a progressive loading of lipids that increases the size of the chylomicrons, which are finally released from the basolateral side of the enterocyte and then secreted into the lymphatic vessels to join the circulation [16,17]. When ApoB-48 is not sufficiently associated with lipids, it is degraded by the ubiquitin–proteasome system [15]. Thus, circulating ApoB-48 concentrations are closely related to the metabolic state and the absorptive capacity of the enterocytes [18,19].

Therefore, we made the hypothesis that circulating ApoB-48 concentrations can be used as a biomarker to assess intestinal absorption efficiency in SBS patients. This study’s aim was to evaluate the relationship between ApoB-48 levels and clinical and biological data, as well as PN dependence, in a cohort of SBS patients.

## 2. Materials and Methods

Patients: This retrospective study was conducted on adult patients with SBS who required PN in an approved center for IF and HPN at the Hospital Beaujon in France. We recruited those who had been diagnosed with a prolonged acute or chronic condition from January 2015 to December 2020 in day hospital or scheduled hospitalization as part of the follow-up. Patients who had active neoplasia, received an alternative treatment for chronic intestinal failure (a reverse intestinal loop, intestinal transplantation, or GLP-2 analogs), or in an unstable/acute condition (such as resection surgery <3 months) were excluded. They were informed about the use of their serum for research purposes, as well as their clinical and biological data. Dietary intake was assessed by a qualified dietitian using a 3-day food recall record. Caloric intake per PN was expressed as the ratio of total calories provided by the PN (kcal/day) to the patient’s body weight in kg. The length of the remaining bowel after the resection was measured by the surgeons during the surgery. Thirty-eight normolipidemic healthy adults (women n = 23) aged 19 to 52 years were recruited to determine fasting ApoB-48 concentrations and to serve as a control group (Generepol, NCT00773201).

Biological measurements: Blood samples were collected in the morning on an empty stomach (8 to 10 h fasting). Cobas 8000 (Roche Diagnostics, Basel, Switzerland) was used to measure albumin, glucose, total cholesterol (T-Ch), triglycerides (TG), HDL-cholesterol (HDL-Ch), C-reactive protein (CRP), and creatinine. LDL-cholesterol (LDL-Ch) was calculated according to the Friedewald formula. After routine blood sampling, serum was stored at −80 °C until analysis. Estimated glomerular filtration rate (GFR) was calculated using the modified MDRD formula (Modification of Diet in Renal Disease). Citrulline was assessed by high-performance liquid chromatography (HPLC). Leptin was measured by Human Leptin ELISA (BioVendor kit, Brno, Czech Republic) and high-molecular-weight adiponectin by Lumipulse G1200 (Fujirebio, Tokyo, Japan).

ApoB-48 quantification: The concentration of ApoB-48 was determined using an ApoB-48 CLEIA Fujirebio kit on a Lumipulse G1200 system (Fujirebio, Tokyo, Japan) and expressed in µg/mL. A pretreatment buffer solution was used to dilute the samples prior to dosing and to dissociate apoB48 from chylomicrons and chylomicron remnants. A second solution containing antibody-coated ferrite particles captured apoB-48 molecules. A second antibody (alkaline-phosphatase-conjugated anti-ApoB-48) converted the chemiluminescent substrate into a signal proportional to the concentration of ApoB-48.

PN dependence ratio (PNDR): The severity of the intestinal failure was assessed using PN dependence. The PN dependence ratio (PNDR) was expressed as the percentage of total daily energy provided by PN divided by the resting energy expenditure (REE) as calculated by the Harris and Benedict equation and taking into account physical activity [20].

Statistics: Statistical analyses were performed with GraphPad Prism software (Prism, version 9.1.0; GraphPad, San Diego, CA, USA). The Shapiro–Wilk test was used to verify the normality of the parameters studied. A Pearson or Spearman test was conducted to assess the correlations according to the distribution of the variables. Results are expressed in median [Q1, Q3]. Comparisons between study groups were analyzed using a Mann–Whitney or Kruskal–Wallis test, followed by Dunn’s post hoc tests. The Hanley and McNeil method was used to compare receiver operating characteristic (ROC) curves.

## 3. Results

### 3.1. Patient Characteristics

Eighty-eight patients were selected among the patients with SBS followed at our expert center and for whom we had sufficient serum in our biobank. Fifty-one SBS patients were analyzed according to our selection criteria. The clinical and biological characteristics of the study population are shown in Table 1. Fifteen patients (29%) had an end-jejunostomy (type I), 25 (49%) had a jejuno–colonic anastomosis (type II), and 11 (22%) had a jejuno–ileal anastomosis (type III). In this cohort, causes of SBS with IF were mesenteric ischemia (n = 25, 49%), surgical complications (n = 8, 15.7%), Crohn’s disease (n = 5, 9.8%), trauma (n = 5, 9.8%), radiation enteritis (n = 4, 7.8%), and motility disorders (n = 4, 7.8%). All subjects were beyond 3 months after their last surgery; 35 patients were PN-dependent with a PN caloric intake of 20 [11.6, 28.2] kcal/kg/day, and 16 were weaned off PN at the time of the study. A comparison of these two groups revealed that weaned-off PN patients were significantly older and had longer remnant small bowel length than PN-dependent patients. Sex ratio, BMI, time between surgery, blood sampling, and the percentage of the remaining colon were similar between these two groups. We collected the daily calorie intake in a subset of the cohort (n = 4 weaned PN and n = 12 dependent PN), but we did not note any significant difference between these two groups. No significant difference was found between weaned-off PN and PN-dependent patients for serum levels of triglycerides, total cholesterol, LDL-cholesterol, estimated glomerular filtration rate, albumin, prealbumin, CRP, glucose, leptin, and HMW adiponectin. However, we found significantly lower levels of HDL-cholesterol (*p* < 0.01) in the PN-dependent group (Table 1).

### 3.2. Relationships between ApoB-48 Concentrations and Clinical and Biological Parameters in SBS Cohort

We measured serum ApoB-48 in the SBS cohort, and we showed that fasting serum ApoB-48 concentrations were positively correlated with triglycerides (*p* < 0.05), total cholesterol (*p* < 0.01), and HDL-cholesterol (*p* < 0.01), negatively correlated with HMW adiponectin (*p* < 0.05) but not related with prealbumin, CRP, leptin, or fasting glucose (Table 2). Kidney function estimated by the GFR was not correlated with ApoB-48 concentrations (r = −0.148, *p* = 0.3) but inversely correlated with citrulline (r = −0.540, *p* = 0.0006).

**Figure 1:**
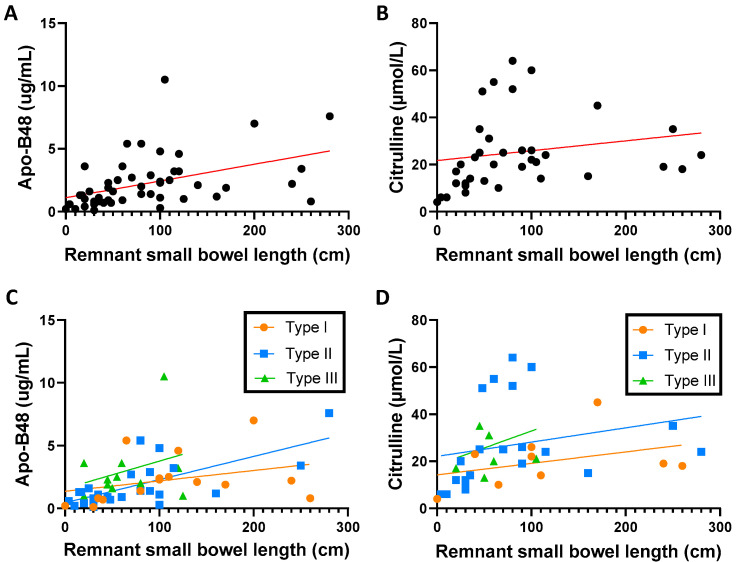
(**A**) Correlation between remnant small bowel length and apolipoprotein B-48 in the study population (Spearman r = +0.55, *p* < 0.0001, n = 51); (**B**) correlation between remnant small bowel length and citrulline in the study population (Spearman r = +0.46, *p* = 0.004, n = 37); (**C**) correlation between remnant small bowel length and concentration of apolipoprotein B-48 regarding the type of anastomosis (Spearman test, type I (r = +0.47, *p* = 0.078, n = 15), type II (r = +0.58, *p* = 0.002, n = 25), and type III (r = +0.13, *p* = 0.69, n = 11)); (**D**) correlation between remnant small bowel length and citrulline regarding the type of anastomosis (Spearman test, type I (r = +0.27, *p* = 0.48, n = 9), type II (r = +0.62, *p* = 0.003, n = 21), and type III (r = +0.36, *p* = 0.44, n = 7)): ApoB-48, apolipoprotein B-48.

We found a significant correlation between serum ApoB-48 concentrations and remnant small bowel length (n = 51, r = 0.546, *p* < 0.0001) in the overall SBS cohort. This correlation was significant in the group of patients with type II anastomosis (n = 25, r = 0.581, *p* < 0.01) but not in the patients with type I (n = 15) or in those with type III anastomosis (n = 11) (Figure 1A,C).

As expected, levels of fasting citrulline were also lower in the PN-dependent group than in the PN-weaned-off group (*p* < 0.05) (Table 1). Fasting citrulline concentrations were correlated with remnant small bowel length (n = 37, r = 0.463, *p* < 0.01) (Figure 1B), and, like ApoB-48, only in the group of SBS patients with type II anastomosis (n = 21, r = 0.615, *p* < 0.01), whereas the correlation was not significant in type I and type III SBS patients (Figure 1D).

### 3.3. ApoB-48 Is Lower in SBS PN-Dependent Patients than in PN-Weaned-Off Patients and Healthy Subjects

**Figure 2:**
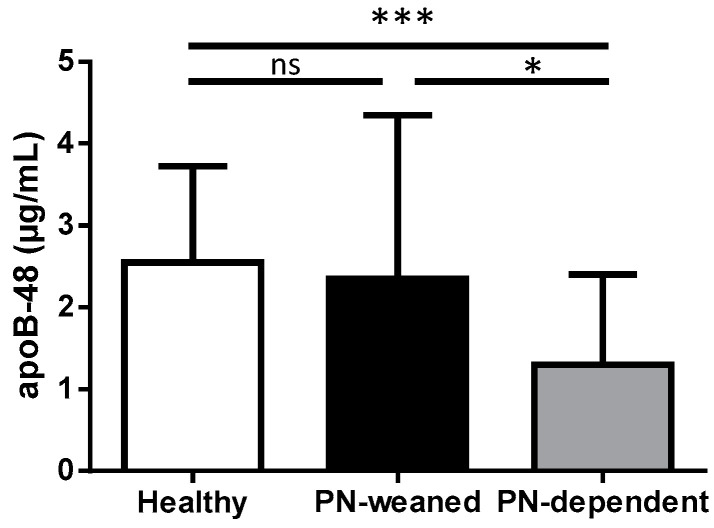
Fasting apolipoprotein B-48 levels in healthy subjects (n = 38), SBS PN-weaned-off patients (n = 16), and SBS PN-dependent patients (n = 35). Data are represented as median and interquartile range; the asterisks indicate a significant difference between groups (Kruskal–Wallis test followed by Dunn’s post hoc test, * *p* < 0.05; *** *p* < 0.001; ns, *p* > 0.05): ApoB-48, apolipoprotein B-48.

We compared fasting serum ApoB-48 in PN-dependent and PN-weaned-off patients with SBS and healthy subjects. A significant difference was found between the three groups (*p* < 0.001). A Wilcoxon–Mann–Whitney test showed that fasting ApoB-48 levels were significantly lower in PN-dependent SBS patients compared with the PN-weaned-off SBS group (*p* < 0.01) and with the healthy subjects (*p* < 0.0001) (Figure 2 and Table 1). Interestingly, levels of fasting ApoB-48 were not significantly different between healthy subjects and PN-weaned-off patients with SBS (2.55 [2.0,3.7] vs. 2.35 [1.5–4.4] µg/mL, *p* > 0.05) (Figure 2).

### 3.4. Fasting ApoB-48 Is Correlated with PNDR

**Figure 3:**
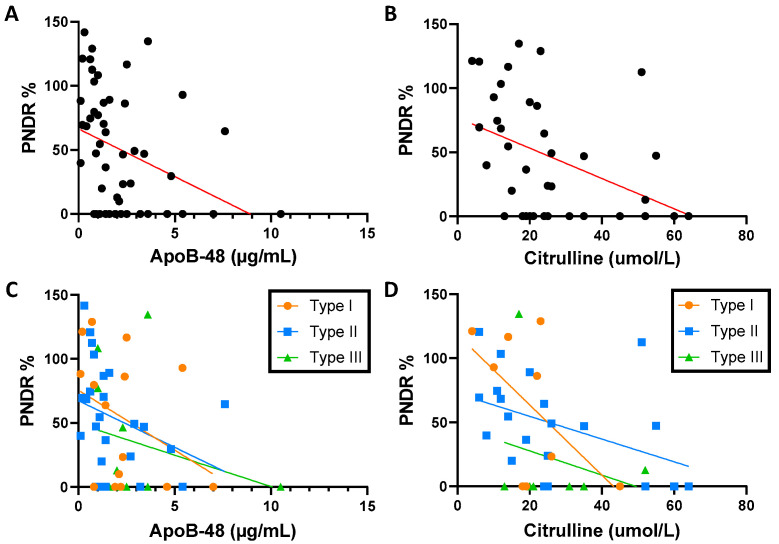
(**A**) Correlation between PNDR and apolipoprotein B-48 (Spearman r = −0.44, *p* = 0.001, n = 51); (**B**) correlation between PNDR and citrulline (Spearman r = −0.49, *p* = 0.002, n = 37); (**C**) correlation between PNDR and concentration of apolipoprotein B-48 according to the type of anastomosis (Spearman test, type I (r = −0.33, *p* = 0.02, n = 15), type II (r = −0.46, *p* = 0.02, n = 25), and type III (r = −0.31, *p* = 0.22, n = 11)); (**D**) correlation between PNDR and concentration of citrulline according to the type of anastomosis (Spearman test, type I (r = −0.39, *p* = 0.28, n = 9), type II (r = −0.54, *p* = 0.01, n = 21), and type III (r = +0.04, *p* = 0.70, n = 7)): PNDR, parenteral nutrition dependence ratio; ApoB-48, apolipoprotein B-48.

To investigate whether fasting ApoB-48 may reflect intestinal absorptive capacities, we examined the relationship between ApoB-48 and PN dependence ratio (PNDR). As shown in Figure 3, PNDR was significantly and negatively correlated with fasting ApoB-48 levels in the overall SBS population (n = 51, r = −0.437, *p* = 0.001) (Figure 3A) and in SBS patients with type II anastomosis (n = 25, r = −0.461, *p* = 0.02) but not in those with type I and type III anastomosis (Figure 3C). Similar relationships were found between PNDR and fasting citrulline in the overall SBS population (n = 37, r = −0.488, *p* = 0.002) (Figure 3B) and in SBS patients with type II anastomosis (n = 21, r = −0.544, *p* = 0.01) (Figure 3D).

**Figure 4:**
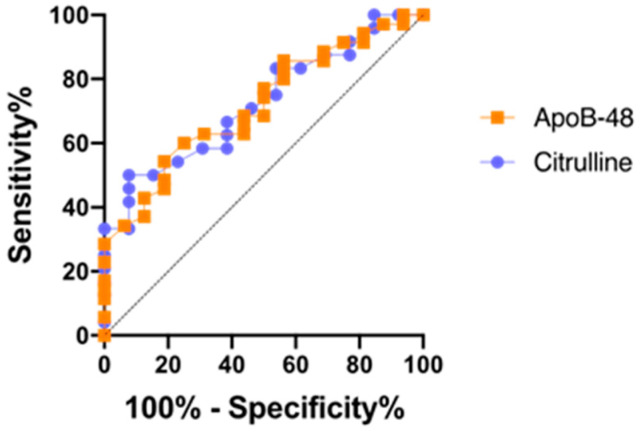
ApoB-48 and citrulline ROC in predicting PN dependence. The Hanley and McNeil method was used for the ROC curve comparison. Apo-B480: AUC= 0.718 (95% IC 0.575–0.861; *p* < 0.05); citrulline: AUC= 0.724 (95% IC 0.561–0.888; *p* < 0.05); (z-score = −0.0584; *p* (two-tailed) = 0.953).

ROC curves were generated for ApoB-48 and citrulline to discriminate PN-dependent patients from weaned-off PN patients. The area under the curve was 0.718 (95% IC 0.575–0.861; *p* < 0.05) for ApoB-48 vs. 0.724 (95% IC 0.561–0.888; *p*< 0.05) for citrulline. No significant difference was found when comparing the two ROC curves of ApoB-48 and citrulline (Figure 4).

## 4. Discussion

To our knowledge, this is the first study showing in patients with SBS that concentrations of ApoB-48 were correlated with residual small bowel length and degree of PN dependence (*p* < 0.01). Among the study population, a positive correlation was found only in the group of patients with jejuno–colonic anastomosis. This subgroup yielded the shortest residual small intestine. These results are consistent with those of the STEPS study [14], which included an equivalent number of patients with SBS per anatomic subtype. The authors of the STEPS study suggested that the shorter the residual small intestine, the stronger the correlation between citrulline and residual bowel length, and that this may also depend on the presence of the colon. They further showed that the use of changes in citrulline concentrations to monitor patients treated with GLP-2 agonists is questionable. In contrast, a negative correlation between citrulline and the severity of enteropathies such as celiac disease and Crohn’s disease has been described, and a weak to no correlation between citrulline and intestinal absorption has been reported [21,22,23,24,25]. It underlines the need for more effective markers, not only for patient follow-up but also for assessing therapeutic response.

Several factors may modulate circulating ApoB-48. Previous studies showed that ApoB-48 levels are regulated by gut peptides such as GLP-1 and GLP-2. Both hormones are synthesized by enteroendocrine cells in the distal small intestine and colon in response to nutrient stimulation [26,27] and increase following intestinal resection [28,29]. However, these enteropeptides have opposite effects on triglyceride-rich lipoproteins and ApoB-48 levels. GLP-2 has been shown to stimulate the release of presynthesized ApoB-48 from the enterocyte [30], whereas GLP-1 agonists significantly reduce the postprandial increase in triglycerides and ApoB-48 [31]. A reduction in ApoB-48 synthesis and an increase in its catabolism could explain this GLP-1 effect [32]. These features make ApoB-48 a potential test companion for monitoring the physiopathology and treatment of SBS patients.

In the literature, high ApoB-48 concentrations have been associated with metabolic diseases, such as obesity, metabolic syndrome, and type 2 diabetes [33,34], and are now identified as a risk factor for coronary artery disease [35]. In our SBS cohort, the lack of correlation between fasting ApoB-48 and BMI or blood glucose is likely related to the metabolic characteristics of the SBS patients, who were neither obese (BMI between 20 and 28 kg/m^2^) nor diabetic. In addition, unlike citrulline, whose levels increase in kidney failure [36], ApoB-48 levels were not related to kidney function in our cohort.

We found positive correlations between ApoB-48 and both triglycerides and cholesterol levels, which is consistent with previous studies showing strong relationships between fasting ApoB-48 and triglycerides or cholesterol levels in healthy adults and adolescents [37,38]. In the literature, high fasting ApoB-48 concentrations are related to the increased secretion rate of intestinal ApoB-48 [39] and/or to liver-impaired ApoB-48 lipoparticle clearance [40]. Although we had no patients with dyslipidemia in our cohort, ApoB-48 levels should be interpreted cautiously in SBS patients when dyslipidemia is acknowledged.

It has been previously shown that ApoB-48 must be associated with lipids to be stabilized and not degraded by the ubiquitin–proteasome system [15]. Therefore, we were surprised to find circulating levels of ApoB-48 in patients with exclusive PN. This result suggests that lipid uptake at the apical side of the enterocyte is not the only way to stabilize ApoB-48. Indeed, it was demonstrated that nonesterified plasma fatty acids can be taken up at the basal pole of enterocytes to synthesize triglycerides, which are then incorporated into chylomicrons [41,42]. In PN-dependent SBS patients without oral nutrition, it can be hypothesized that basal uptake of plasma fatty acids by enterocytes is sufficient to stabilize a small amount of ApoB-48.

An interesting result is that fasting ApoB-48 concentrations were significantly lower in PN-dependent SBS patients compared with weaned patients. In addition, considering a subset of the cohort, we found no significant difference in oral intake between weaned and dependent PN patients despite the difference in ApoB-48 levels between the two groups. These results suggest that patients with higher ApoB48 exhibit higher lipid transfer, whether due to higher oral lipid intake or greater intestinal absorption efficiency.

Finally, we found that PN-weaned-off SBS patients had similar fasting ApoB-48 concentrations compared with healthy subjects, which may reflect the achievement of intestinal absorption efficiency and, thus, PN independence. Altogether, these results allow us to propose ApoB-48 not only as a marker of the enterocyte mass but also to assess enterocyte absorptive function in SBS patients regardless of oral nutrition. This should be tested in a larger prospective study by longitudinal assessment of ApoB-48 levels during the adaptation phase and in patients receiving GLP-2 agonists.

In addition, the automated method for ApoB-48 quantification is an important asset, allowing a rapid and reliable assay, thus facilitating the use of this marker. In fact, apolipoprotein IV, another apolipoprotein synthesized by enterocytes, has shown a stronger correlation with small intestinal residual length than citrulline. However, its measurement is still subject to manual techniques, which hinders its use as a follow-up marker [43].

Altogether, our study suggests that ApoB-48 can be a useful marker to evaluate the functionality of the remnant small bowel in patients with SBS. Measuring ApoB-48 can help monitor intestinal adaptation and follow-up patients treated with GLP-2 agonists. These treatments are very expensive, with heterogeneous responses among patients, and for which no markers are yet available to evaluate the effectiveness [44,45,46,47].

We know that the retrospective nature of this study constitutes a limit to our presentation. In order to validate the role of ApoB-48 in the assessment of intestinal energy absorption capacity, a prospective study should be conducted in SBS patients to correlate ApoB-48 levels to the gold standard bomb calorimetry assay and determine the cut-off values. These studies should also assess the impact of potential factors influencing ApoB-48 concentrations, such as oral lipid intake. It would also be interesting to assess the relationship between inflammatory markers, including cytokines, and concentrations of this apolipoprotein in order to rule out a possible impact. Finally, most of the SBS patients included in this study underwent jejuno–colonic anastomosis, and few patients presented an end-jejunostomy or jejunoileal anastomosis. A study of larger groups may be needed to confirm whether ApoB-48 concentrations are significantly correlated with residual small bowel length in type I and type III SBS groups.

## 5. Conclusions

This study showed that circulating ApoB-48 was strongly associated with the enterocyte mass and individual PN requirement. ApoB-48 could be proposed as a marker of intestinal absorptive function and, therefore, be of interest as a biomarker in the management of patients with SBS. Further studies are needed to compare whether circulating ApoB-48 and citrulline are redundant or complementary markers in assessing intestinal mass, absorptive intestinal function, or monitoring nutritional requirements.

## Figures and Tables

**Table 1:** Clinical and biological characteristics of SBS patients in the study population and according to PN dependence.

	Study Population (n = 51)	PN Weaned (n = 16)	PN Dependent (n = 35)	*p*-Value *
Gender (M/F)	25/26	10/6	15/20	
Age (years)	55.4 [46–68]	60 [53–71]	54 [39–67]	<0.05
BMI (kg/m^2^)	21.3 [20–23]	22.3 [21–28]	20.8 [20–23]	ns
Remnant small bowel length (cm)	70 [30–110]	102.5 [56–158]	48 [25–100]	<0.01
Remnant colon (%)	70 [0–90]	85 [0, 100]	65 [0–80]	ns
Stomy	16	6	10	-
Duration from surgery (years)	2.55 [1.7–9.3]	3.54 [0.7–10.6]	2.55 [1.9–9.2]	ns
Oral intake (kcal/day)	2450 [1775, 2681] (n = 16)	2350 [1825, 2800] (n = 4)	2450 [1625, 2681] (n = 12)	ns
**Anastomosis type**				
End-jejunostomy (type I)	15	5	10	-
Jejunocolic anastomosis (type II)	25	5	20	-
Jejunoileal anastomosis (type III)	11	6	5	-
**SBS etiology**				
AMI/VMI	25	8	17	-
Radiation enteritis	4	0	4	-
Crohn’s disease	5	3	2	-
Surgical complications	8	3	5	-
Trauma	5	1	4	-
CIPO primary/idiopathic	4	1	3	-
**Biochemical measurements**				
Triglycerides (mmol/L)	1.21 [0.9–1.8]	0.99 [0.7–1.8]	1.22 [0.9–1.9]	ns
Total cholesterol (mmol/L)	3.10 [2.6–4.1]	3.59 [3.1–4.2]	2.8 [2.3–4.1]	ns
LDL-cholesterol (mmol/L))	1.60 [1.0–2.3]	1.78 [1.3–2.6]	1.55 [1.0–2.3]	ns
HDL-cholesterol (mmol/L)	0.88 [0.6–1.3]	1.19 [0.8–1.6]	0.70 [0.5–1.0]	<0.01
Creatinine (mmol/L)	74 [64–105]	78.5 [65–105]	73 [61–105]	ns
GFD (ml/min/1.73 m^2^)	88 [61–109]	82 [62–102]	88 [51–113]	ns
Albumin (g/L)	37.7 [32–41]	39.9 [37–44]	35.3 [30–40]	ns
Prealbumin (g/L)	0.25 [0.2–0.3]	0.27 [0.2–0.3]	0.24 [0.2–0.3]	ns
CRP (mg/L)	1 [1–3.8]	1.5 [1–2.5]	1 [1–4]	ns
Glucose (mmol/L)	5.10 [4.7–5.8]	4.80 [4.4–5.2]	5.30 [4.8–6]	ns
Leptin (ng/mL)	4.0 [2–7]	4.89 [1.5–7.2]	3.58 [1.8–11]	ns
Adiponectin (ng/mL)	5.41 [4–11.5]	4.72 [3.9–5.4]	9.09 [4.6–12.8]	ns
ApoB-48 (µg/mL)	1.60 [0.8–2.9]	2.35 [1.5–4.4]	1.30 [0.7–2.4]	<0.01
Citrulline (µmol/L)	21.0 [14–33]	25.0 [20–49]	18.0 [11–26]	<0.05

Results are expressed in median [Q1, Q3]; * *p*-value was calculated using Mann–Whitney test comparing the weaned-off parenteral nutrition and parenteral nutrition dependent patients (ns: *p* > 0.05): AMI/VMI, acute mesenteric ischemia; BMI, body mass index; ApoB-48, apolipoprotein B-48; CRP, C-reactive protein.

**Table 2:** Correlation coefficients relating ApoB-48 concentrations and biological measurements in the study population and according to PN dependence.

Correlation Matrix		ApoB-48	
	Study Population (n = 51)	PN Weaned (n = 16)	PN Dependent (n = 35)
Triglycerides	0.326 *	0.488	0.329
Total cholesterol	0.452 **	0.513	0.351 *
LDL-cholesterol	0.290 *	0.445	0.123
HDL-cholesterol	0.380 **	0.207	0.438 *
Creatinine	0.153	0.06	0.295
GFR	−0.148	−0.240	−0.190
Albumin	0.271	−0.148	0.318
Prealbumin	−0.077	−0.051	−0.071
CRP	0.258	0.462	0.272
Glucose	−0.094	−0.280	0.005
Leptin	0.160	0.119	0.276
HMW adiponectin	−0.427 *	−0.245	−0.539 *

Asterisks indicate a significant correlation between groups (Pearson or Spearman test, * *p* < 0.05, ** *p* < 0.01): ApoB-48, apolipoprotein B-48; HMW adiponectin, high-molecular-weight adiponectin; GFR, glomerular filtration rate.

## Data Availability

Data are available upon reasonable request to the corresponding author.
